# Study of Cysteine-Rich Protein 61 Genetic Polymorphism in Predisposition to Fracture Nonunion: A Case Control

**DOI:** 10.1155/2015/754872

**Published:** 2015-12-10

**Authors:** Sabir Ali, Syed Rizwan Hussain, Ajai Singh, Vineet Kumar, Shah Walliullah, Nazia Rizvi, Manish Yadav, Mohammad Kaleem Ahmad, Abbas Ali Mahdi

**Affiliations:** ^1^Department of Orthopaedic Surgery, King George's Medical University, Lucknow, Uttar Pradesh 226 018, India; ^2^Molecular Cell Biology Lab, Biochemistry, King George's Medical University, Lucknow, Uttar Pradesh 226 003, India

## Abstract

*Background*. Many factors are responsible for this impaired healing, especially in long bones, but a possible genetic predisposition for the development of this complication remains unknown till now. In the present study, we aim to examine the CYR61 gene polymorphism in fracture nonunion patients and the correlation with clinical findings.* Materials and Methods*. We performed SNP analysis of the CYR61 gene in 250 fracture nonunion patients and 250 healthy subjects were genotyped in this hospital-based case control study, and 56 cases were further evaluated for mRNA expression of CYR61 by real-time quantitative reverse-transcription PCR.* Results*. CYR61 gene TT, TG, and GG genotype frequencies of total fracture nonunion cases were 41.6%, 49.2%, and 9.20% and 54.4%, 39.2%, and 6.40% in healthy controls. Heterozygous TG genotype was found statistically significant in fracture nonunion cases compared with that in controls, whereas homozygous mutant GG genotype was not found significant. Moreover, we found that TG + GG genotypes were significantly different in serum expression of CYR61 mRNA when compared with cases (TT genotypes).* Conclusions*. Our result signifies that genotype of CYR61 affects the mRNA expression and acts as a risk factor that could synergistically increase the susceptibility of a patient to develop fracture nonunion.

## 1. Introduction

Fractures are a common orthopaedic problem, mostly in long bones. Of all long bones, shaft of tibia is one of the commonest bones to get fractured with a relatively higher incidence of impaired healing at the fracture site due to lesser soft tissue coverage, being a subcutaneous bone on anterior aspect [1, 2]. The mentioned reasons account for a high rate of tibial nonunions amounting for 2–10% of all tibial fractures leading to significant patient morbidity [3–6]. Apart from the reasons mentioned, other factors contributing to fracture nonunion are as follows: soft tissue damage, inadequate mechanical stability, open fractures, administration of pharmacological agents, such as NSAIDs, smoking, and so forth [7, 8]. Adequacy of vascular supply to the fracture site is an essential prerequisite for healing process, whereas inadequate vascularity results in delayed/nonunion [9].

Fracture shaft of tibia still frequently develops nonunion despite all optimized multifactorial conditions. This tendency of fracture may reflect the role of some major genetic components involved in the process of bone regeneration and fracture repair. There are studies which support genetic variability as one of the significant associations contributing to the process of bone regeneration as well as fracture healing rate [10, 11]. Although associations with genetic variability are much talked about, their potential role in predisposition to fracture impairment still remains unknown and needs to be elucidated.

The CYR61 (cysteine-rich protein 61) gene is located on chromosome 1p22. This is an important molecule which was shown to participate in a number of key cellular processes including cell differentiation, adhesion, migration, proliferation, wound healing, and angiogenesis [12–14]. The CYR61 gene is key signalling molecules involved in angiogenesis that is prerequisite for the initial process of fracture healing. Many times due to improper angiogenesis at the fracture site, impaired healing may occur. As per our knowledge, present study is the first to suggest the possible effect of a functional polymorphism (promoter region, rs3753793) in the CYR61 gene on expression of CYR61 mRNA in nonunion tibial fracture cases.

The role of the single nucleotide polymorphism (SNP) involving CYR61 gene in fracture healing, if proved, may open new horizons for innovations in this field with an addition to our armamentarium to deal with complications associated with fracture healing. The association if established will add genetic predisposition as a new risk factor for impaired bone healing. It can also in the future be used as an important prognostic tool for early identification of patients at risk of impaired fracture healing.

This study is probably the first of its kind carried out in north Indian population to evaluate the association between CYR61 gene polymorphism ((T → G) gene polymorphism) and fracture nonunion in shaft of tibia.

## 2. Materials and Methods

### 2.1. Samples Collection

A total of 250 fracture shafts of tibia with nonunion cases and 250 healthy subjects were selected for the study. The study was carried out in the Department of Orthopaedic Surgery (OPD), King George's Medical University, Lucknow. The Institutional Review Board and Ethics Committee of King George's Medical University, Lucknow, approved this study and it was carried out during January 2011 to December 2014. Before enrolment, each subject's written informed consent was obtained in response to a fully written and verbal explanation of the nature of study.

### 2.2. Inclusion and Exclusion Criteria

Cases were defined as subjects of either sex within age group of 18–45 years with fracture nonunion shaft of tibia defined as bone healing with Radiographic Union Score for Tibial Fractures (RUST) < 7 by the end of 6th month, along with clinical indication of nonunion like pain, abnormal mobility with presence of no transmitted movements at site of fracture [15]. Controls were defined as otherwise normal subjects of similar age group. Exclusion criteria included children and patients with a known systemic inflammatory disease, osteoporosis and other metabolic bone diseases, pathological fractures and subsequent nonunions, and hypertrophic and infected nonunions [16].

### 2.3. Genomic DNA Isolation

Venous blood was collected in 0.5 M EDTA vial and stored at −80°C. Genomic DNA extraction for molecular genetic studies was performed using the commercially available extraction kit (Bangalore Genei, India) and was stored at −80°C. DNA concentration was measured with a Nanodrop ND-100 Spectrophotometer (Thermo Fisher Scientific Inc., Wilmington, DE).

### 2.4. Analysis of CYR61 Gene (T → G) Polymorphism by PCR Amplification

**Figure 1:**
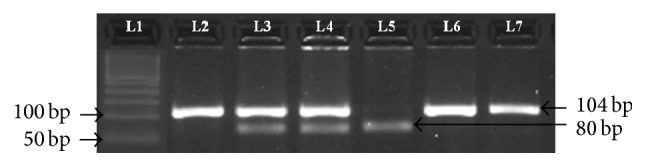
3% agarose gel analysis of CYR61 (T → G) gene polymorphism. Lane 1: 50 bp ladder. Lanes 2, 6, and 7: TT genotype 104 bp. Lanes 3 and 4: TG genotype 104, 80, and 24 bp. Lane 5: GG genotype 80, 24 bp.

The CYR61 (T → G) gene polymorphism (rs3753793) was analysed by the polymerase chain reaction (PCR) followed by restriction fragment length polymorphism (RFLP). Genomic DNA was amplified (Applied Biosystems, Veriti, Singapore) using the following PCR conditions: 95°C for 6 min, 36 cycles at 95°C for 55 s, 57°C for 45 min, 72°C for 1 min, and finally 72°C for 10 min. The primers used for amplification of the CYR61 (T → G) gene polymorphism were as follows: forward primer** 5**′**-CTT GCC TCT CAC CTT CGC TGT TAA-3**′ and reverse primer** 5**′**-GTC GTT TTG TTT GGT GAT GCG A-3**′ [17]. Amplification was performed with 25 *μ*L PCR reaction mixture containing 100 ng template DNA, 10 pmol of each primer, and 2x PCR master mixes (Fermentas, Germany). Amplification success of samples was monitored by 3% agarose gel electrophoresis. Thereafter the PCR products were subjected to digestion by* KspA* I enzyme (Fermentas, Germany) to screen for the CYR61 (T → G) gene polymorphism. The enzymatic mixture contained 1 *μ*L restriction enzyme, 1 *μ*L 10x buffer, 6 *μ*L PCR products, and 2 *μ*L distilled water; the mixture was incubated overnight at 37°C for digestion. The digested product was electrophoresed on 3% agarose gel electrophoresis at 80 volts for one hour. In cases with CYR61 (T → G) gene polymorphism, an undigested 104 bp band showed wild-type TT genotype, while two bands of 80 and 24 bp confirmed mutant GG genotype and three bands of 104, 80, and 24 bp were detected in the heterozygous TG genotype [17] (Figure 1). Among the controls, the genotype frequency of the polymorphism was confirmed by Hardy-Weinberg equilibrium (*P* > 0.05, shown in Table 2). The quality control testing was performed using GAPDH. Also all the RFLP genotyping was later confirmed using the sanger sequencing method in subgroups (homozygous, wild type, heterozygous, and variant homozygous).

### 2.5. RNA Extraction and Analysis of Real-Time PCR

To further detect the correlation between the CYR61 mRNA levels and genotypes polymorphism in vivo, fracture nonunion specimens (whole blood) were obtained from 56 cases with different genotypes. Total RNA was isolated using the extraction kit (Fermentas, Germany) and was stored at −80°C. RNA concentration was measured with a Nanodrop ND-100 Spectrophotometer (Thermo Fisher Scientific Inc., Wilmington, DE). An aliquot of the total RNA (15 *μ*L) from each sample was reverse transcribed into single-strand cDNA using an oligo(dT) [18, 19]. The primers used for PCR amplification were** 5**′**-CCT GTC CGC TGC ACA CCA GC-3**′ and** 5**′**-GGA GAG CGC CAG CCT GGT CA-3**′ for CYR61 and** 5**′**-GAA ATC CCA TCA CCA TCT TCC AGG-3**′ and** 5**′**-GAG CCC CAG CCT TCT CCA TG-3**′ for glyceraldehydes-3-phosphate dehydrogenase (GAPDH). The expression of CYR61 relative to GAPDH RNA was determined using the method as explained [20].

### 2.6. Sequence Analysis

All samples selected for real-time PCR (*n* = 56) were also characterized by automated sequencing. The PCR product of each sample was first purified and then submitted in 25 *μ*L quantity with 10 picomoles of appropriate primer. The sequencing was performed by an automated direct DNA sequencing technique, which incorporates fluorescently labelled dideoxynucleotides during cycle sequencing and separates the resulting products by capillary electrophoresis for detection on an ABI 3730XL DNA Analyser (Applied Biosystems, USA). Multiple alignment and sequence analysis were done using BLAST (Basic Local Alignment Search Tool), BioEdit, FinchTV, and AutoAssembler Software (Applied Biosystems, USA). Sequences obtained were aligned using the BioEdit software with normal sequences taken from Genbank and examined for the presence of polymorphism (Figure 3).

### 2.7. Statistical Analysis

The significance of this study was evaluated by Chi-square test. Odds ratio (OR) was calculated as an estimate of relative risk of having disease according to the relative frequency of different genotypes among the cases as well as the controls. The association between the polymorphism and fracture nonunion was estimated by odds ratios (ORs) and their 95% confidence intervals (CIs), which were calculated by unconditional logistic regression. *P* value was considered significant at <0.05. The value was expressed in mean ± SD (Standard Deviation). By considering power of 80% with minimum expected difference between the two means of 6.7, the sample size of *n* = 86 was obtained. However, in the present study, we enrolled 250 cases as well as controls.

## 3. Results

In our study we recruited 250 fracture nonunion cases, including 147 males and 103 females, with age ranging from 18 to 45 years. The demographic and the baseline data were compared as in Table 1. All the cases and controls were successfully genotyped by PCR-RFLP. The average CYR61 (T → G) TT, TG, and GG genotype frequencies in total fracture nonunion cases were 41.6%, 49.2%, and 9.20% and 54.4%, 39.2%, and 6.40% in healthy controls, respectively. The frequency of CYR61 (T → G) gene polymorphism and statistical analysis of the cases and controls are shown in Table 2. The observed CYR61 (T → G) high expression mutant G allele frequency was 33.8% in fracture nonunion cases and frequency was 26.0% in healthy controls (Table 2).

In this study among 250 cases and 250 controls, we found that TG genotype was present among 123 nonunion cases and 98 controls, the GG genotype was present in 23 fracture nonunion cases and 16 healthy controls, and the TT genotype was present among 104 fracture nonunion cases and 136 healthy controls (Table 2). The heterozygous TG genotypes were more prevalent in fracture nonunion cases compared with healthy controls and difference between cases and controls was statistically significant (OR = 1.64, 95% CI = 1.13–2.37, and *P* = 0.010), whereas homozygous GG genotype was not significant. The frequency of mutant G allele in CYR61 (T → G) was statistically significant in fracture nonunion cases compared with that in healthy controls (OR = 1.45, 95% CI = 1.10–1.90, and *P* = 0.008) suggesting that individual G allele was associated with fracture nonunion cases (Table 2).

**Figure 2:**
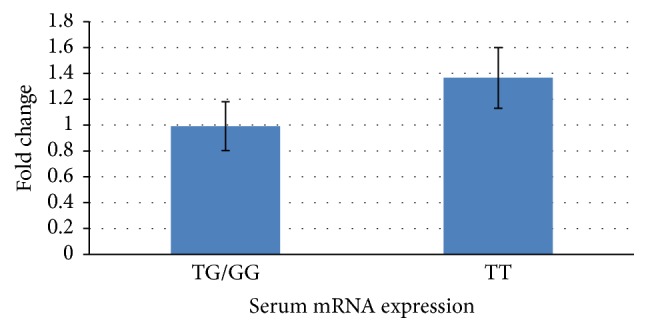
CYR61 gene transcript in fracture nonunion patients detected by real-time quantitative reverse-transcript PCR. The frequency distributions of the TT, TG, and GG genotypes were 25, 28, and 3, respectively. The fold changes were 1.16 for TT (±0.54) and 0.855 for TG/GG (±0.35), which were standardised against GAPDH.

**Figure 3:**
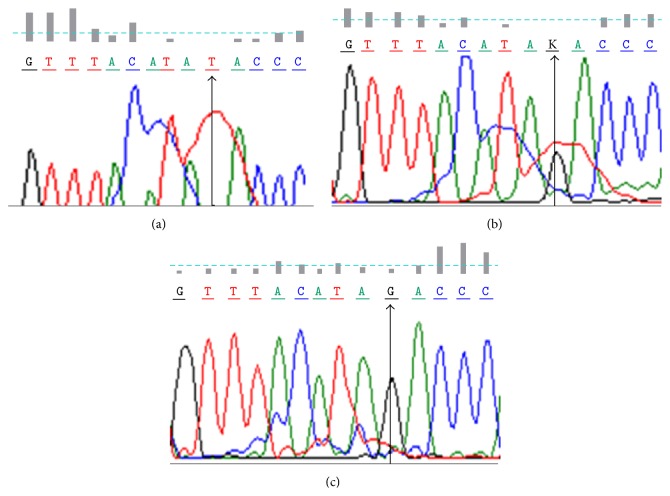
Chromatograms of three cases showing (arrow) the three genotypes of the single nucleotide polymorphism found in CYR61 gene. (a) Genotype TT homozygous wild type. (b) Genotype TG heterozygous (K = T/G). (c) Genotype GG homozygous mutant.

Further the association of CYR61 gene polymorphism with CYR61 mRNA expression in 56 fracture nonunion cases was analysed. The effect of these three genotypes on CYR61 mRNA expression was evaluated by real-time quantitative reverse-transcription PCR. CYR61 quantification showed that homozygous wild type of the TT genotype was with significantly increased mRNA levels when compared with homozygous/heterozygous mutant of the GT/GG genotype (*P* = 0.01; *t* = 2.5521; CI = −0.54460 to −0.06540; Figure 2).

## 4. Discussion

This study was carried out on north Indian population with the aim of investigating and assessing whether a single nucleotide polymorphism (SNP) in the promoter region of the CYR61 gene is associated with fracture nonunion risk in shaft of tibia or not.

As is well known, reasons for fracture nonunion in shaft of tibia are multifactorial, and even with favourable conditions, nonunion is relatively common in shaft of tibia. This observation forces us to widen our vision to look into a possible genetic factor responsible for impaired fracture healing. On observing various genotypes, we concluded that TT genotype was significantly associated with increased rate of fracture healing progression compared with GG and TG genotypes. Further analysis revealed that fracture nonunion cases with homozygous wild type of the TT genotype had significantly increased mRNA levels contrary to homozygous/heterozygous variants of the GG and TG genotypes. From above observation, it can be inferred that fracture healing outcome therefore is possibly attributed to genetic variations among patients. Manigrasso and O'Connor in their study established significant contribution of genetic variability in the process of bone regeneration and in their experiment on mice strains, they also demonstrated the effect of genetic variability on the length of each stage of fracture healing and the overall healing rate [10].

Another study reported a genetic polymorphism at functional promoter fragment of the human CYR61 gene on CYR61 mRNA expression in cases of nonunion fracture shaft tibia [21]. The above-mentioned effect is probably due to the fact that this polymorphism may affect the transcriptional activity of CYR61 gene. As per our knowledge, present study is the first to suggest the possible effect of functional polymorphism in the CYR61 gene on expression of CYR61 mRNA in nonunion cases. The possible explanation for this altered expression may be the polymorphism in the promoter region of CYR6, which may affect the transcriptional activity. However, further studies are needed to better explore the regulatory mechanisms of genetic variants that further affect mRNA expression.

In the present study, we observed a statistically significant effect of the G allele of CYR61 for developing fracture nonunion, suggesting that the polymorphism may affect the progression of bony union, as the G allele is associated with decreased mRNA level of CYR61 gene, which may be one of the causes of fracture nonunion. The basic concept of the present study is supported by study of Tao et al. (2013), in which they observed that the G allele of rs3753793 (TGþGG) had significantly lower risk of prostate cancer by downregulating the mRNA expression of CYR61 when compared with the TT genotype [17]. Thus, this study proves that G allele of CYR61 may affect the mRNA expression. Further, apart from polymorphic study, expressional study by Hadjiargyrou et al. (2000) showed upregulation of CYR61 mRNA and protein expression after fracture promoted healing by inducing the angiogenesis process, which is prerequisite for any fracture healing [21]. Lienau et al. also found an upregulation of the CYR61 protein expression during the early phase of healing especially in the chondrogenesis process [22].

In conclusion, we provide the first evidence supporting the genetic effect of CYR61 gene polymorphism on risk of fracture nonunion in north Indian population. In this study the author also supposed that the homozygous/heterozygous mutant G allele may affect the optimum angiogenesis process at the initial phase of healing, which leads to increased fracture nonunion risk followed by decrease in the mRNA expression of CYR61 in related bone fracture nonunion cases. The author also realised further multicentric studies with large sample size to strengthen the obtained results.

## Figures and Tables

**Table 1:** Demographic details of fracture nonunion patients and controls.

Characteristics	Cases (*n* = 250)	Controls (*n* = 250)	*P* value
Age	35.92 ± 4.94	34.37 ± 5.21	0.923
Male	58.8% (*n* = 147)	64.4% (*n* = 161)	0.578
Female	41.2% (*n* = 103)	35.6% (*n* = 89)	0.438
Site of fracture, left	107 (42.8%)	—	—
Site of fracture, right	143 (57.2%)	—	—
Haemoglobin (Hb)	10.15 ± 1.22	—	—
Albumin	3.53 ± 0.25	—	—
Ferritin	142.54 ± 33.8	—	—

**Table 2:** Genotype and allele frequencies of CYR61 (T → G) gene polymorphism in fracture nonunion patients and healthy controls.

CYR61 (T → G) genotyping	Cases (*n* = 250)	Controls (*n* = 250)	*P* value	Odds ratio	95% CI	Chi-square
TT	104 (41.6%)	136 (54.4%)	—	—	—	—
TG	123 (49.2%)	98 (39.2%)	0.010^*∗*^	1.64	1.13–2.37	6.50
GG	23 (9.20%)	16 (6.40%)	0.099	1.88	0.94–3.73	2.70
TG + GG	146 (58.4%)	114 (45.6%)	0.005^*∗*^	1.67	1.17–2.38	7.70
T	331 (66.2%)	370 (74.0%)	—	—	—	—
G	169 (33.8%)	130 (26.0%)	0.008^*∗*^	1.45	1.10–1.90	6.88

^*∗*^Significant value.

## References

[1] Reed L. K., Mormino M. A. (2008). Distal tibia nonunions. *Foot and Ankle Clinics*.

[2] Patel M., McCarthy J. J., Herzenberg J. Tibial Nonunion.

[3] Marsh D. (1998). Concepts of fracture union, delayed union, and nonunion. *Clinical Orthopaedics and Related Research*.

[4] Praemer A., Furner S., Rice D. P. (1992). *Musculoskeletal Conditions in the United States*.

[5] Sarmiento A., Gersten L. M., Sobol P. A., Shankwiler J. A., Vangsness C. T. (1989). Tibial shaft fractures treated with functional braces. Experience with 780 fractures. *The Journal of Bone & Joint Surgery—British Volume*.

[6] Phieffer L. S., Goulet J. A. (2006). Delayed unions of the tibia. *The Journal of Bone & Joint Surgery—American Volume*.

[7] Brinker M. R., Browner B. D., Jupiter J. P., Levine A. M., Trafton P. G. (2003). From nonunions: evaluation and treatment. *Skeletal Trauma: Basic Science, Management, and Reconstruction*.

[8] Giannoudis P. V., MacDonald D. A., Matthews S. J., Smith R. M., Furlong A. J., De Boer P. (2000). Nonunion of the femoral diaphysis. The influence of reaming and non-steroidal anti-inflammatory drugs. *The Journal of Bone & Joint Surgery—British Volume*.

[9] Hausman D. B., DiGirolamo M., Bartness T. J., Hausman G. J., Martin R. J. (2001). The biology of white adipocyte proliferation. *Obesity Reviews*.

[10] Manigrasso M. B., O'Connor J. P. (2008). Comparison of fracture healing among different inbred mouse strains. *Calcified Tissue International*.

[11] Jepsen K. J., Hu B., Tommasini S. M. (2007). Genetic randomization reveals functional relationships among morphologic and tissue-quality traits that contribute to bone strength and fragility. *Mammalian Genome*.

[12] Bork P. (1993). The modular architecture of a new family of growth regulators related to connective tissue growth factor. *FEBS Letters*.

[13] Jay P., Bergé-Lefranc J. L., Marsollier C., Méjean C., Taviaux S., Berta P. (1997). The human growth factor-inducible immediate early gene, CYR61, maps to chromosome 1p. *Oncogene*.

[14] Perbal B. (2001). NOV (nephroblastoma overexpressed) and the CCN family of genes: structural and functional issues. *Molecular Pathology*.

[15] Whelan D. B., Bhandari M., Stephen D. (2010). Development of the radiographic union score for tibial fractures for the assessment of tibial fracture healing after intramedullary fixation. *Journal of Trauma and Acute Care Surgery*.

[16] Dimitriou R., Carr I. M., West R. M., Markham A. F., Giannoudis P. V. (2011). Genetic predisposition to fracture non-union: a case control study of a preliminary single nucleotide polymorphisms analysis of the BMP pathway. *BMC Musculoskeletal Disorders*.

[17] Tao L., Chen J., Zhou H. (2013). A functional polymorphism in the CYR61 (IGFBP10) gene is associated with prostate cancer risk. *Prostate Cancer and Prostatic Diseases*.

[18] Hussain S. R., Naqvi H., Mahdi F., Bansal C., Babu S. G. (2013). KIT proto-oncogene exon 8 deletions at codon 419 are highly frequent in acute myeloid leukaemia with inv(16) in Indian population. *Molecular Biotechnology*.

[19] Babic A. M., Kireeva M. L., Kolesnikova T. V., Lau L. F. (1998). CYR61, a product of a growth factor-inducible immediate early gene, promotes angiogenesis and tumor growth. *Proceedings of the National Academy of Sciences of the United States of America*.

[20] Livak K. J., Schmittgen T. D. (2001). Analysis of relative gene expression data using real-time quantitative PCR and the 2^−ΔΔ*C*_T_^ method. *Methods*.

[21] Hadjiargyrou M., Ahrens W., Rubin C. T. (2000). Temporal expression of the chondrogenic and angiogenic growth factor CYR61 during fracture repair. *Journal of Bone and Mineral Research*.

[22] Lienau J., Schell H., Epari D. R. (2006). CYR61 (CCN1) protein expression during fracture healing in an ovine tibial model and its relation to the mechanical fixation stability. *Journal of Orthopaedic Research*.

